# Bone Mineral Density in the Noninstitutionalized Elderly: Influence of Sociodemographic and Anthropometric Factors

**DOI:** 10.1155/2016/4946593

**Published:** 2016-04-05

**Authors:** Ellen Cristina de Sousa e Silva Araujo, Valéria Pagotto, Erika Aparecida Silveira

**Affiliations:** ^1^Faculty of Nutrition, Federal University of Goiás, Rua 227 Qd. 68 s/n°, Setor Leste Universitário, 74605-080 Goiânia, GO, Brazil; ^2^Faculty of Nursing, Federal University of Goiás, Rua 227 Qd. 68 s/n°, Setor Leste Universitário, 74605-080 Goiânia, GO, Brazil; ^3^Graduate Health Sciences Program, Faculty of Medicine, Federal University of Goiás (UFG), Rua 235 c/1a s/n, Setor Leste Universitário, 74605-020 Goiânia, GO, Brazil

## Abstract

*Objective*. Analysis of bone mineral density (BMD) in the elderly and its associated factors according to sex.* Methods*. A cross-sectional study is presented herein, with a random sample of 132 noninstitutionalized elderly people. Individuals who did not use diuretics were excluded. BMD was obtained from examination of total body densitometry and its association with sociodemographic variables, lifestyle, anthropometric, and body composition was verified.* Results*. Mean BMD for men was 1.17 ± 0.12 g/cm^2^ and for women was 1.04 ± 0.11 g/cm^2^. Higher education was associated with higher BMD values in men (*p* < 0.05). There was a reduction in BMD in the age group 75–79 years of age in women and over 80 years of age in men (*p* < 0.05). Underweight was associated with significantly low BMD for both sexes (*p* < 0.01), while normal weight was associated with low BMD in women (*p* < 0.001).* Discussion*. The elderly with low schooling and in older age groups are more probable to also present low BMD. Lower levels of body mass index also indicated towards low BMD.

## 1. Introduction

The bone mineral content (BMC) reaches its peak concentration before the third decade of life [1], and two factors can contribute to reduction: a low peak of bone mass and/or significant loss of BMC during adult life. Low bone mineral density (BMD) exposes the bone to fractures when these are submitted to small trauma, which could compromise the quality of life of the elderly, besides increasing the probability of morbidities and mortality [2].

In Brazil, 15.1% of women and 12.8% of men have already suffered low-impact fractures due to low BMD [3]. Only in 2011, 37,967 Brazilian elderly were admitted to the hospital because of femur fractures [4]. Low BMD and the consequent fractures have negative impacts on health services as assistance is onerous and requires specialized teams; for the elderly, the impact is more pronounced as life quality decreases and mortality increases [5].

The aging process, however, is not the only determinant factor for issues with bone mineral density [6]. In scientific literature, there are several modifiable risk factors described for low BMD, such as low weight, abusive consumption of alcohol, use of tobacco, level of physical activity, and low income [7, 8]. Anthropometric and body composition parameters can also be associated with low BMD, but findings remain controversial. Some studies disagree that adiposity indices protect low BMD [9, 10], while other authors consider these parameters as beneficial for low BMD [11].

Due to these controversial findings on risk factors for BMD [6, 10] as well as the great impact of bone fragility issues on elderly individuals, this study has the objective of analyzing BMD in community elderly and its association with sociodemographic variables, lifestyle, body composition, and anthropometric parameters, by sex.

## 2. Materials and Methods

A cross-sectional study is presented herein, within the framework of research project* Projeto Idosos/Goiânia* [12] (Elderly Project/Goiânia), which evaluated 418 elderly individuals randomly selected by multiple-stage sampling. Data were collected at the residence of the elderly by trained interviewers and anthropometrists, who utilized standardized anthropometric measurement techniques. The methodological details on sample calculations and sampling were described in a previous publication [12].

Twelve elderly people were randomly selected from the initial sample for evaluation of BMD. Dual-energy X-ray absorptiometry (DXA, previously DEXA) was carried out in accordance with the following eligibility criteria: no use of diuretics, body weight under 100 kg, and height under 1.90 m. Data collection for this subsample was accomplished in a specialized clinic, with a trained team constituted by nutrition academics, nutritionists, and a nurse. The elderly were contacted by phone to schedule the appointment for data collection and to inform on the preparation for the tests: fasting, no use of diuretics, and no physical exercise on the day of the appointment.

The following variables were analyzed: age (classified in five-year periods), living arrangements (e.g., with partner), per capita income (classified in quartiles), education level (in years), tobacco smoking, excessive consumption of alcohol, physical activity, weight, height, body mass index (BMI), body fat percentage (BFP), waist circumference (WC), waist-hip ratio (WHR), bone mineral content (BMC), bone area, and bone mineral density (BMD).

Excessive alcohol consumption was classified in accordance with the Brazilian Cardiology Society, with a daily limit of 30 g of ethanol for men and 15 g for women [13]. Physical activity was classified according to the short version of the* International Physical Activity Questionnaire* [14], and the elderly were divided into three categories: sedentary, moderate practice of activity, and vigorous practice of activity. Consumption of tobacco was classified into three categories: the elderly that have never smoked, ex-smokers, and smokers.

Weight was measured by a digital portable Tanita® scale model UM 080W, with 150 kg capacity and 0.1 kg precision. Height was measured with a wooden square and inelastic measuring tape fixed to a wall, 50 cm from the ground, with alignment verified by a plumb line. For measurement of circumferences, a flexible and inelastic measuring tape was used, with a 0.1 cm precision. WC was classified as adequate when it was <80 cm in women and <94 cm in men, enlarged when it was ≥80 cm and ≤88 cm in women and ≥94 cm and ≤102 cm in men, and very enlarged when it was >88 cm in women and >102 cm in men [15].

The waist-hip ratio was calculated from WC and hip circumference (HC), to verify the risk of cardiovascular diseases, as classified by Bray [16] for individuals over the age of 60. Moderate risk was considered when WHR > 0.8 for women and 0.9 for men. The BMI was calculated by dividing the body mass by the height square and classified according to Lipschitz [17] and as recommended by Silveira et al., [18] where values under 22 kg/m^2^ indicated malnutrition and values equal or over 27 kg/m^2^ indicated overweight.

DXA was utilized to estimate BMD and body fat percentage; the body scanner equipment measures the composition of body compartments. The equipment was regularly calibrated, and the values of BMC, bone area, and BMD (equal to BMC divided by bone area) were obtained.

The database was built utilizing the program EPIDATA® version 3.1, with double entry to avoid inconsistencies. Data were analyzed by STATA/SE 12.0. All analyses were stratified by sex. The differences between averages were analyzed by the *t*-test, ANOVA, or Kruskal-Wallis, with a 5% significance level. The *t*-test was applied to compare binomial variables, and when three or more averages required comparison, ANOVA was applied to normally distributed variables and the Kruskal-Wallis test was applied when the variables were not assumed to be normally distributed.

In the bivariate analysis, the association between BMD and the remaining variables was evaluated by brute linear regression. The variables presenting *p* < 0.20 in the bivariate analysis were inserted in the multiple-hierarchy linear regression model in two levels: first level, sociodemographic variables, and second level, lifestyle, anthropometric, and body composition variables. Those variables that presented *p* ≤ 0.05 were included in the adjusted model.

The project was approved by the Research and Ethics Committee of the Federal University of Goiás (031/2007) and all participants signed free informed consent forms.

## 3. Results

Finally, 132 elderly people were evaluated: 80 women (61%) and 52 men (39%), with average age 70 ± 6.4 years. The average values of weight, height, BMI, BMC, and bone area were higher in men. BMD presented normal distribution. The average BMD in women was 1.04 ± 0.11, and in men this value was 1.17 ± 0.12 with a statistically significant difference (*p* = 0.000) (Table 1).

A reduction in average values of BMD (Table 2) was verified in women as age increased (*p* = 0.019); in men, average BMD increased with the education level (*p* = 0.011).

Bivariate analysis revealed association between BMD and education level (*p* = 0.011), BMI (*p* = 0.000), and WC (*p* = 0.025) for men. For women, associations were verified with age (*p* = 0.019), BMI (*p* = 0.000), BFP (*p* = 0.000), and WC (*p* = 0.000) (Tables 2 and 3). The multiple-hierarchy linear regression model considered the following variables for men: age, education level, tobacco smoking, BMI, BFP, WC, and WHR (Table 4). For women, the included variables were age, BMI, BFP, and WC (Table 5).

The multivariate model explained 44% of variability of BMD in men and 42% of variability in women (Tables 4 and 5). The variables that remained associated with BMD in men after adjustments in the multivariate model were age (≥80 years of age, *p* = 0.012), 0–4 years of education (*p* = 0.018), 5–7 years of education (*p* = 0.005), and low weight (*p* = 0.002) (Table 4). For the female gender, the variables that remained associated with BMD were age (75 to 79 years of age, *p* = 0.044; and ≥80 years of age, *p* = 0.043), low weight (*p* = 0.000), and eutrophy (*p* = 0.000) (Table 5).

## 4. Discussion

Reduction in BMD is a gradual and silent process, and the occurrence of risk factors can accentuate this process, increasing the negative outcomes affecting the health of the elderly. Access to BMD evaluation tests would help identify the issue and plan interventions. However, this is a high cost test, which hinders early diagnosis and monitoring, especially in medium- and low-income countries of Latin America and Asia. The current study allowed for the identification of the BMD profile in the elderly population that lives in community settings, besides identifying some factors associated with its reduction: education level, advanced age, and body mass index. These results can contribute to the planning of more specific actions for the health of the elderly, helping prevent fractures and decrease in life quality, in accordance with the risk factors identified. Few studies have utilized a gold standard test (DXA) to evaluate the body composition and BMD in the elderly of both genders [19, 20].

There are some divergences on the measurement locations or full body BMD in studies evaluating the prevalence of low BMD. Studies that utilize full body BMD are important as they enable the investigation of the bone mass situation throughout the skeleton and how it is affected by risk factors. Therefore this is also a contribution of the research presented herein, as full body BMD was assessed, contributing to the discussion of these aspects [19–21]. However, some caution as the exclusion of the elderly who did not use diuretics has been taken to ensure the quality of the examination.

The average full body BMDs obtained herein were higher than the values found in a European study [22], but similar to the values found in studies with men over the age of 50 [20] and the female elderly in Brazil [19]. Regarding BMC, the elderly studied herein presented higher averages than the Chinese population and lower values than the Caucasian elderly of the United States, for both sexes [9]. Chinese lifestyle is a protection factor for BMD reduction, and probably genetic aspects have a greater influence on BMD [22]. Race and genetic aspects are important factors in BMD, as studies report that Caucasians present lower BMD when compared to the African race [22].

The reduction of BMD with the progression of age is coherent with other BMD studies [7, 8]. This finding is corroborated by the reduction in BMD after the bone mass peak is achieved, approximately at the third decade of life, in consequence of the loss of minerals and reduction in the synthesis capacity of the bone tissue [23]. A significant reduction was observed after 75–79 years of age in the female gender and after 80 years of age for the male gender (five years after women). This difference can be related to alterations in the concentration of sexual hormones that are connected to the bone tissue metabolism, such as estrogen [24].

Unfavorable social conditions seem to have contributed to low BMD [8, 25]. In the present study, the level of education remained to be associated with BMD, which agrees with other findings [25], also pointing to better levels of education as a protection factor against BMD loss in both genders. The level of education can be a determining factor for access to better work and leisure conditions, health services, and decision-making regarding health treatments and life habits [8]. All of these aspects are linked to bone health.

There are results indicating that abusive consumption of alcohol is a risk factor for BMD reduction [26]. However, no association was verified herein between the excessive ingestion of alcohol and BMD reduction, which could be related to the low frequency of alcohol consumption. It is still much discussed whether the consumption of alcohol is a risk factor or protection factor for BMD. Studies indicate that, besides the amount of alcohol ingested, the quality of the alcohol can affect this association [27]. This could be related to the beneficial influence of nonalcoholic compositions on the health of bones [27].

There is yet much discussion on the association between the smoking of tobacco and BMD, but some studies have established associations towards low BMD risk [7, 28], while another study did not verify any association [6], corroborating the findings presented herein.

The present study also corroborates other findings regarding the association between the body mass index and higher average BMD [10, 25]. The relationship between adiposity parameters and BMD is still widely discussed [10, 11]. Discussions on the increase of BMD along with body weight approach two aspects: effect of the hormones related to obesity [10] and/or a skeletal adaptation process resulting from increase of mechanic load on the bone [11]. In this hypothesis, the bone cells act as mechanic sensors detecting mechanic loads on the bone [11]. In the present study, low weight in both genders and eutrophy in the female gender were associated with a more pronounced reduction in BMD, which is against the mechanical load and skeletal adaptation hypothesis. However, total or abdominal adiposity measurements such as body fat percentage and waist circumference were not associated, in both genders, after multivariate analysis adjustments. These findings can suggest that the body mass, that is, the mechanical load, acts with more influence to maintain the bone integrity rather than body fat or adiposity.

Environmental factors and lifestyle accumulate throughout life and influence BMD, and therefore the variables collected in the present study referred to the current habits of the elderly. Nevertheless, sociodemographic aspects and even body composition factors usually do not suffer great variations throughout life. Therefore the cross-sectional character of the study is in accordance with the established objectives and provides important information. It must be highlighted that there was special methodological care regarding the selection of the sample, training of the team, pilot study, and standardization of anthropometric measurements to guarantee the quality of the data presented.

It is concluded that higher educational levels were associated with higher average BMD in elderly men. A reduction in BMD was observed with age, especially after 75 years of age in women and after 80 years of age in men. Lower BMD was associated with low weight in both genders and with eutrophy in women. Body composition and adiposity variables, consumption of alcohol, smoking habits, and practice of physical activity were not associated with BMD. It is recommended that future studies are carried out to verify the association between these variables and BMD since youth, in a longitudinal study.

A possible unfolding of this research is that these results are useful to identify the elderly with higher risks of low BMD and, from these data, plan strategies for the promotion and protection of bone health. Since BMD diagnosis tests entail high costs and are not accessible to the general population, identification of risk factors in the elderly can contribute to health triage and vigilance and to carrying out prevention actions against the occurrence of adverse events related to low BMD, such as falls and fractures. It is suggested that efforts are directed to public policies and social projects destined to the elderly population to promote an adequate nutritional state (as low weight is a risk factor for low BMD).

## Figures and Tables

**Table 1:** Bone mineral density, nutritional parameters, and age of the elderly, by sex. Goiânia, GO, 2009. *n* = 132.

Variables	Sample (*n* = 132)	Men (*n* = 52)	Women (*n* = 80)	*p* value^*∗*^
Age (years)	70.01 ± 6.40	70.50 ± 6.68	69.69 ± 6.23	0.478
Weight (kg)	67.78 ± 14.09	71.72 ± 13.04	65.22 ± 14.23	0.009
Height (m)	1.59 ± 0.08	1.67 ± 0.07	1.54 ± 0.05	0.000
BMI (kg/m^2^)	26.73 ± 5.19	25.75 ± 4.05	27.38 ± 5.75	0.078
Bone area (cm^2^)	2.04 ± 0.25	2.25 ± 0.20	1.91 ± 0.18	0.000
BMC (kg)	2.26 ± 0.50	2.65 ± 0.45	2.00 ± 0.35	0.000
BMD (g/cm^2^)	1.09 ± 0.13	1.17 ± 0.12	1.04 ± 0.11	0.000

^*∗*^Student's *t*-test.

BMI: body mass index; BMC: bone mineral content; BMD: bone mineral density.

**Table 2:** Bone mineral density in the elderly, by sex, and association with sociodemographic and lifestyle variables, Goiânia, GO, 2009. *n* = 132.

Variables	*n* (%)	Bone mineral density			
		Men		Women	
		Average ± SD	*p* value	Average ± SD	*p* value
Age^*∗*^			0.160		0.019
60–64	26 (19.70)	1.22 ± 0.14		1.06 ± 0.13	
65–69	43 (32.58)	1.88 ± 0.10		1.08 ± 0.09	
70–74	33 (25.00)	1.17 ± 0.11		1.05 ± 0.13	
75–79	18 (13.64)	1.16 ± 0.14		0.99 ± 0.10	
Over 80	12 (9.09)	1.06 ± 0.10		0.93 ± 0.09	
Living with partner^‡^			0.251		0.971
Yes	76 (57.58)	1.18 ± 0.11		1.04 ± 0.09	
No	56 (42.42)	1.13 ± 0.14		1.04 ± 0.13	
Income quartile^*∗*^			0.707		0.225
1st quartile	33 (25.58)	1.03 ± 0.12		1.19 ± 0.10	
2nd quartile	32 (24.81)	1.06 ± 0.10		1.14 ± 0.13	
3rd quartile	32 (24.81)	1.03 ± 0.12		1.14 ± 0.09	
4th quartile	32 (24.81)	1.06 ± 0.11		1.22 ± 0.14	
Years of school^*∗*^			0.011		0.606
0–4	77 (63.11)	1.14 ± 0.11		1.03 ± 0.12	
5–7	22 (18.03)	1.13 ± 0.11		1.00 ± 0.11	
Over 8	23 (18.85)	1.24 ± 0.11		1.05 ± 0.07	
Tobacco use^†^			0.105		0.927
Nonsmoker	64 (48.48)	1.19 ± 0.13		1.04 ± 0.11	
Ex-smoker	54 (40.91)	1.09 ± 0.11		1.06 ± 0.14	
Smoker	14 (10.61)	1.17 ± 0.11		1.04 ± 0.11	
Excessive consumption of alcohol^‡^			0.955		0.4713
No	123 (93.18)	1.17 ± 0.11		1.04 ± 0.11	
Yes	9 (6.82)	1.17 ± 0.16		1.10 ± 0.08	
Physical activity^*∗*^			0.525		0.205
Sedentary	66 (50.00)	1.18 ± 0.13		1.03 ± 0.11	
Moderate activity	31 (23.48)	1.20 ± 0.10		1.08 ± 0.12	
Vigorous activity	35 (26.52)	1.15 ± 0.12		1.04 ± 0.11	

^*∗*^ANOVA; ^‡^ *t*-test, ^†^Kruskal-Wallis.

**Table 3:** bone mineral density in the elderly, per gender, and association with anthropometric and body composition variables. Goiânia, GO, 2009. *n* = 132.

Variables	*n* (%)	Bone mineral density			
		Men		Women	
		Average ± SD	*p* value	Average ± SD	*p* value
BMI (WHO)			0.001^*∗*^		0.0001^*∗*^
Low weight	5 (3.79)	1.13 ± 0.0		0.92 ± 0.14	
Eutrophic	51 (38.64)	1.09 ± 0.09		0.97 ± 0.08	
Overweight	44 (33.33)	1.21 ± 0.11		1.07 ± 0.08	
Obese	32 (24.24)	1.25 ± 0.10		1.13 ± 0.10	
BMI (Lipschitz)			0.000^§^		0.000^§^
Low weight	22 (16.67)	1.08 ± 0.07		0.92 ± 0.10	
Eutrophic	52 (39.39)	1.15 ± 0.11		1.00 ± 0.80	
Overweight	58 (43.94)	1.24 ± 0.11		1.10 ± 0.10	
BFP			0.025^#^		0.000^#^
Adequate	54 (40.91)	1.14 ± 0.13		0.99 ± 0.10	
Augmented	78 (59.09)	1.18 ± 0.11		1.08 ± 0.10	
WC			0.025^§^		0.000^§^
Adequate	33 (25.19)	1.12 ± 0.09		0.94 ± 0.11	
Enlarged	33 (25.19)	1.16 ± 0.14		1.00 ± 0.07	
Very enlarged	65 (49.62)	1.22 ± 0.09		1.09 ± 0.10	
WHR			0.158^#^		0.880^#^
No risk for CNTD	12 (9.16)	1.11 ± 0.10		1.05 ± 0.12	
At risk for CNTD	119 (90.84)	1.18 ± 0.12		1.04 ± 0.11	

^*∗*^Kruskal-Wallis; ^#^Student's *t*-test; ^§^ANOVA; BMI: body mass index; BFP: body fat percentage; WC: waist circumference; WHR: waist-hip ratio; CNTD: chronic nontransmissible diseases.

**Table 4:** Linear regression coefficients (brute and adjusted) for bone mineral density according to sociodemographic, lifestyle, anthropometric, and body composition variables, in elderly men. Goiânia, GO, 2009. *n* = 132.

Variables	*β*_brute_	*p* value^#^	Hierarchic multivariate analysis		
			*β*_Aj_^‡^	*p*-value^#^	*R* ^2^
1st level					0.29
Age					
60–64	1.00		1.00		
65–69	−0.046	0.355	−0.047	0.323	
70–74	−0.047	0.323	−0.030	0.520	
75–79	−0.064	0.274	−0.055	0.320	
Over 80	−0.166	0.013	−0.158	0.012	
Years of school					
0–4	−0.102	0.009	−0.090	0.018	
5–7	−0.115	0.008	−0.120	0.005	
Over 8	1.000	—	1.00		

2nd level					0.44
Use of tobacco^*∗*^					
Nonsmoker	1.000		1.000		
Ex-smoker	−0.025	0.470	0.004	0.896	
Smoker	−0.102	0.063	−0.068	0.221	
BMI (WHO)					
Low weight	−0.115	0.299	−0.089	0.400	
Eutrophic	−0.154	0.001	−0.141	0.003	
Overweight	−0.032	0.461	−0.039	0.376	
Obese	1.00	—	1.00	—	
BMI (Lipschitz)					
Low weight	−0.161	0.000	−0.149	0.002	
Eutrophic	−0.096	0.004	−0.059	0.110	
Overweight	1.00	—	1.00	—	
BFP^*∗*^					
Adequate	−0.044	0.185	0.488	0.246	
Augmented	1.000	—	1.000		
WC^*∗*^					
Adequate	−0.108	0.007	−0.020	0.720	
Enlarged	−0.062	0.116	−0.017	0.718	
Very enlarged	1.000	—	1.000		
WHR^*∗*^					
No risk for CNTD	0.064	0.158	0.059	0.316	
At risk for CNTD	1.000	—	1.000		

^*∗*^Due to loss of statistical significance, was not maintained in the final model of multivariate linear regression (*p* > 0.05).

^#^Linear regression test.

^‡^Adjusted by age and years of school (education level).

**Table 5:** Linear regression coefficients (brute and adjusted) for bone mineral density according to sociodemographic, lifestyle, anthropometric, and body composition variables, in elderly women. Goiânia, GO, 2009. *n* = 132.

Variables	*β*_brute_	*p* value^*∗*^	Multivariate analysis	
			*β*_Aj_^#^	*p* value
Age				
60–64	1.000	—	—	—
65–69	0.020	0.553	0.009	0.756
70–74	−0.012	0.751	−0.008	0.805
75–79	−0.065	0.124	−0.073	0.044
Over 80	−0.122	0.014	−0.085	0.043
BMI (WHO)				
Low weight	−0.202	0.000	−0.120	0.264
Eutrophic	−0.152	0.000	−0.156	0.001
Overweight	−0.058	0.037	−0.042	0.348
Obese	1.00	—	—	—
BMI (Lipschitz)				
Low weight	−0.184	0.000	−0.173	0.000
Eutrophic	−0.096	0.000	−0.084	0.000
Overweight	1.00	—	1.00	—
BFP^*∗*^				
Adequate	0.091	0.000	0.010	0.761
Augmented	1.00	—	1.00	—
WC^*∗*^				
Adequate	−0.146	0.000	−0.070	0.165
Enlarged	−0.086	0.003	−0.05	0.184
Very enlarged	1.00	—	1.00	—

^*∗*^Was not included in the final multivariate linear regression model. ^#^Adjusted by age and BMI.

*R* ^2^ = 0.42.
